# Outdoor food advertising landscape along school-age children’s daily commute routes in Chengdu: implications for childhood obesity prevention

**DOI:** 10.1186/s12889-025-25806-2

**Published:** 2025-12-11

**Authors:** Chunyu Zhao, Chongjun Bi, Min Feng, Jingru Ma, Jingwei Che, Xiaohui Li, Yongsheng Li, Xiaohua Lyu

**Affiliations:** 1 West China School of Public Health and West China Fourth Hospital, Sichuan University, Chengdu, 610041 China; 2 United Nations Children’s Fund (UNICEF), Beijing, China; 3 Chengdu Center for Disease Control and Prevention, Chengdu, China; 4 Health Promotion and Food Nutrition & Safety Key Laboratory of Sichuan Province, Chengdu, China

**Keywords:** Food advertising, Outdoor advertising, Marketing themes, Unhealthy foods, Childhood obesity

## Abstract

**Background:**

The advertising of unhealthy foods has been recognized as a significant contributor to childhood overweight and obesity. In China, childhood obesity has become an escalating public health concern. Despite this, data on outdoor food advertising, one of the most common advertising forms that children encounter in their daily lives is not well documented in China, leaving gaps in an understanding of its exposure, impact and implications for policy interventions. Collaborating with UNICEF China, this study investigated outdoor advertising in 3 key settings (roadside environment around schools, high-rise apartment communities, and public transit systems), aiming to fill these gaps and support a healthier environment across China.

**Methods:**

Multi-stage stratified random sampling methods were utilized to select investigated schools, high-rise apartment communities, and public transit systems in Chengdu. The INFORMAS Outdoor Advertising Protocol guided the selection of the physical routes covered in the investigations, while WHO Nutrient Profile Model for the Western Pacific Region served as the technical basis for categorizing the food items advertised. At the same time, advertising themes in outdoor advertisements of unhealthy foods were identified through qualitative coding and content analysis.

**Results:**

A total of 998 outdoor advertisements were observed around 25 schools, of which 439 (44.0%) were food advertisements. 308 (70·2% of all food advertisements) were for unhealthy foods. In 903 high-rise apartment communities, 2864 distinctive print advertisements and 1174 non-repetitive video advertisements were recorded, with approximately one-third (32·6%&35·5%, respectively) food-related, and over half of food-related advertisements through each medium (52·8%&64·4%, respectively) marketing unhealthy foods. In public transit, 3385 advertisements were collected through 70 bus routes and Metro Line 1 to Line 8, including 232 (6·9%) food advertisements, with 114 (49·1%) for unhealthy foods. Along children’s daily commute routes, sugar-sweetened beverages and alcoholic beverages were the most frequently advertised unhealthy foods. Outdoor food advertisements in Chengdu, especially those for unhealthy foods, are mainly concentrated in central city. Video advertisements for unhealthy foods in residential buildings employed various marketing strategies, with taste appeal, nutrition/health claims, and promotional characters being the most prevalent themes.

**Conclusion:**

School-age children in Chengdu are highly exposed to outdoor advertisements for unhealthy foods, with residential building elevators serving as a major hotspot for such advertising. Strengthening policy oversight of outdoor advertising for unhealthy foods is a crucial measure for preventing childhood obesity.

## Introduction

Over the past four decades, the global childhood obesity rate has significantly increased. Obesity not only adversely affects the physical and mental health of children and adolescents but also serves as a significant risk factor for various chronic diseases in adulthood [1].

Childhood obesity results from a combination of multiple factors, including genetic influences, poor dietary habits, and lack of physical activity [2]. In addition, societal factors play a crucial role in the development of childhood obesity [3]. Strong evidence indicates that prolonged exposure to unhealthy food and beverage advertising may significantly influence children’s food preferences, choices, and consumption behaviors. This, in turn, leads to an increased intake of calories and an imbalanced diet, thereby heightening the risk of obesity and associated chronic non-communicable diseases [4]. The World Health Organization (WHO) recognizes that the marketing and advertising of unhealthy foods, particularly those high in fat, sugar, and salt (HFSS), are significant drivers of increasing childhood overweight and obesity rates [5].

In May 2010, 192 member states of the WHO adopted Resolution WHA63·14 [6], which aims to restrict the marketing of foods and non-alcoholic beverages high in saturated fats, trans fatty acids, added sugars, and/or added salts to children and adolescents on a global scale, so as to reduce the incidence of overweight, obesity, and diet-related non-communicable diseases. Following this, countries and regions such as Chile [7], Quebec (Canada) [8], and Latvia [9] have enacted legislation and various management measures, even elevating the regulation of food advertising on television to a national health security strategy in order to protect children’s health. In response to these regulations, food companies have had to adjust their advertising strategies, focusing more on responsible marketing approaches in less regulated areas. For example, money is being invested in outdoor advertising where regulation is relatively weak, and sports drinks marketed in stadiums are used to convey a “healthy” image through mainstream sporting events. Teenagers generally confuse the need for sports drinks with water, while underestimating their sugar content [10].

In the modern era, outdoor media has become the third-largest advertising media after television and online platforms. Outdoor advertising refers to commercial, public service, and signage advertisements displayed on external surfaces of buildings, municipal infrastructure, outdoor venues, urban rail transit systems, and other public spaces through various formats including text, graphics, video content, digital displays, and physical installations. This category encompasses multiple forms such as billboards, illuminated light boxes, electronic screen ads, building facade ads, vehicle-mounted ads, elevator ads, and aerial advertising systems [11]. Outdoor advertisements are often placed in public spaces such as shopping centers, transportation hubs, and school zones, where children are exposed to them. Globally, existing studies show that outdoor advertising for unhealthy foods is a widespread trend. The study of 21 middle and high income countries shows that almost a quarter of ads across all studies were for food (mean of 22.1%) and the majority of advertised foods were unhealthy (mean of 63%) [12]. Reviews have showed the pervasiveness of unhealthy food advertising around school areas [13, 14]. In addition, public transportation acts as a hub for ads of unhealthy foods. Swedish scholars have found public transit systems to be flooded with ads for ultra-processed foods (often high in fat, salt, and/or sugar) [15]. A study in the U. S. found a positive correlation between food outdoor advertising and obesity. Those who lived in areas with a greater percentage of food ads had increased odds of overweight and obesity. For every 10% increase in food advertising, there was a 1·05 (95% CI 1·003—1·093, *p* < 0·03) greater odds of being overweight or obese, controlling for other factors [16].

Reducing children’s exposure to unhealthy food marketing is an important strategy to prevent obesity, and policies to limit outdoor advertising of unhealthy foods are reasonable and feasible. However, formulating such policies is complex due to various factors, including defining outdoor advertising, defining unhealthy foods, identifying age groups of children that need protection, limiting marketing strategies, and determining the types of media to be restricted. The lack of data on these technical elements may lead to policy design flaws, so it is essential to accumulate international data to refine policies that protect children.

China has become a major area affected by the obesity crisis. According to the *Report on the Nutrition and Chronic Disease Status of Chinese Residents (2020)*, nationwide, the prevalence of overweight and obesity among children and adolescents aged 6–17 was 19%. By the end of 2023, the obesity rate for children and adolescents aged 6–17 in Chengdu, a densely populated southwest city where obesity had not been as prevalent as in some other cities in the country, had reached 24·9%. The governor of Chengdu is committed to addressing the pressing issue of childhood obesity by protecting children from an obesogenic food environment. A 5-year partnership has been established with UNICEF China, with endorsement from the National Health Commission. This collaboration has underscored the need to introduce policies and regulations to ‘clean’ the outdoor environment and reduce the impact of unhealthy food advertising on children. However, without concrete data and a consistent monitoring mechanism, the formulation of these policies and regulations has not yet been feasible. In addition, with the characteristics of China, many children live in high-rise residential communities, and elevator ads are a unique form of outdoor advertising that must be considered. This study, commissioned by UNICEF China and the Chengdu CDC, focuses on the “point-to-point” daily commuting routes of school-age children in Chengdu, specifically the areas surrounding schools, high-rise apartment communities, and public transit systems. It aims to map the exposure of children to outdoor food advertising in Chengdu. To facilitate international communication and data comparison, the study refers to the International Network for Food and Obesity/Non-communicable Diseases Research, Monitoring and Action Support (INFORMAS) Outdoor Advertising Protocol and WHO Nutrient Profile Model^1^ for the Western Pacific Region. The goal is to fill data gaps and provide scientific evidence to support the development of relevant policies and regulatory measures by the appropriate authorities.

## Methods

### Scope of surveyed outdoor advertisements

The below scope is adjusted and expanded based on the INFORMAS protocol, with modifications rooted in China’s legal framework, children’s actual activity patterns, and findings from pilot studies.

#### Outdoor advertisements around schools

This includes ads directly visible on the exterior of food-related establishments such as stores, restaurants, and shopping malls, as well as those displayed along roads, hanging or posted on telephone poles, banners suspended across streets, wall ads along sidewalks, and posters on buildings.

#### Outdoor advertisements in high-rise apartment communities

This encompasses both video and print ads within high-rise apartment communities. Video ads include those displayed on projection screens, electronic screens, and elevator TV monitors in waiting areas, and ads on the interior walls and doors of the elevator. Print ads mainly consist of framed posters in waiting areas, elevator interior posters, and adhesive posters on elevator doors.

#### Outdoor advertisements through public transit systems

This category includes surface bus ads and those displayed in the subway system. Surface bus ads encompass vehicle body ads, in-vehicle TV ads, posters inside buses, and billboards at bus stops. Subway advertising, on the other hand, takes various forms within the subway system. These include ads on mobile TVs, subway handles and doors within subway cars, exterior vehicle body paintings or branded trains. Additionally, subway stations commonly feature creative displays on lightboxes, pillar wraps in elevator areas, pedestrian tunnel billboards, escalator posters, and ads along tunnel walls.

### Sampled sites

#### Schools

Multi-stage stratified random sampling was used. In the first stage of sampling, Wuhou District and Longquanyi District were selected as representatives of the central areas of Chengdu and surrounding areas, respectively. In the second phase of sampling, based on the list of day school institutions provided by Chengdu Municipal Education Bureau’s official website, we used a random number table to select 7 primary schools and 6 secondary schools from 70 primary schools and 47 secondary schools in Wuhou District, 6 primary schools and 6 secondary schools each from 53 primary schools and 40 secondary schools in Longquanyi District, totaling 25 primary and secondary schools as survey sites.

#### High-rise apartment communities

A multi-stage stratified random sampling method was implemented. In the first stage, five central urban districts (Wuhou District, Jinjiang District, Qingyang District, Jinniu District, and Chenghua District) and three surrounding districts (Shuangliu District, Longquanyi District, and Wenjiang District) in Chengdu were selected. During the second stage, a random number table was employed to sample residential buildings across eight administrative districts. The calculated sample size ranged from 683 to 1,008 units. Ultimately, 903 high-rise apartment complexes across these eight districts were included in the study.

The sample content calculation formula is as follows [17]:

$$\mathrm n=\frac{\mathrm{Z}^{2}_{{\upalpha}/2}\times\left[\mathrm\uppi\times\left(1-\mathrm\uppi\right)\right]}{\mathrm{E}^2}$$

among which n: sample size;

Z_α/2_: statistical quantity, Z_α/2_ = 1.96;

E: Sampling error, E = 0.03;

π: Probability value, referring to the exposure rate of food ads on TV in China, it ranges from 19.7% to 38.3% [18, 19].

#### Public transit systems

To select bus routes, a 10% random sample was drawn from the total number of surface bus routes passing through schools and residential buildings in Chengdu, using a random number table. This resulted in 70 bus routes being included in the study. The line covers chengdu’s Jinjiang District, Wuhou District, Chenghua District, Longquanyi District, Jinniu District and other administrative areas, with a total length of 1,018.6 km. Additionally, all metro lineswere incorporated into the analysis.

### Coding

The INFORMAS Outdoor Advertising Protocol’s standard template [20] was used to record ads. For each advertisement, information was coded on:

1. The distance of the food/beverage advertisement from school (within 250 m).
2. The size of the advertisement (small (21 cm × 30 cm—1·3 m × 1·9 m), medium (> 1·3 m × 1·9 m—2·0 m × 2·4 m), large (≥ 2 m × 2·5 m)).
3. The height of the advertisement (low (0–0·8 m), medium (0·8–1·8 m), high (≥ 1·8 m)).
4. The setting of the advertisement (food shop, road, building, bus shelter, train station, cart/stall).
5. The type and position of the advertisement (billboard, poster, free-standing, painted, digital/LED, store merchandising).
6. Whether the subject of the advertisement was for single or multiple foods and beverages.
7. Major food category (core/healthy, non-core/unhealthy, miscellaneous).
8. Minor food category (e. g. sugar-sweetened beverages, alcoholic beverages, savoury food snacks, healthy food snacks, water, baby foods, baby and toddler milk), divided into 26 food categories.

### Data collection

The data were formally collected from July to September 2023.

#### Outdoor advertisements around schools

Using Gaode Map, the entrance of each selected school served as the center, around which a 250-m radius was drawn. Within this defined area, all accessible roads were mapped as survey routes. Two investigators inspected all outdoor advertisements along each survey route, documenting details such as the distance of food and beverage ads from the school, advertisement size, location, type, and duration of advertisement display using photographs.

#### Outdoor advertisements in residential buildings

For every selected community, two investigators conducted the surveys together. They visited randomly selected residential buildings and recorded all video and print ads within accessible elevator areas using digital cameras. They collected data including advertisement size, height, food and beverage types, and the duration of food ads.

#### Outdoor advertisements through public transit systems

Each pair of investigators conducted surveys along the selected bus or metro lines. At each designated bus/metro station, they proceeded in the direction of travel, recording continuous footage with mobile phones. They documented all ads inside and outside the bus or subway car, as well as those at the stations.

### Qualitative coding and content analysis of marketing themes

A qualitative codebook was further refined based on prior studies [21, 22]. Every collected unhealthy food advertisement was reviewed by two coders (unlike advertising collectors) to assess whether marketing strategies were employed, with specific types of strategies identified. Each advertisement could utilize multiple marketing themes. To ensure the reliability and consistency of the two coders, the research team conducted a consistency testing by randomly sampling 100 photos of unhealthy food ads and 10 video ads. The two coders independently encoded the ads, and their results were compared for consistency. The Kappa value was greater than 0·7, indicating a high level of reliability, allowing the coding process to proceed.

The codebook classified the marketing themes of unhealthy food ads into eight categories:

Premium offers (e. g. free gifts, toys, discounts, competitions and vouchers);

Child-oriented features (e. g. use of a child model or childlike character (even in the presence of an adult), the words ‘ child’ or ‘ kid’ or placement next to a school or playground);

Promotional characters (e. g. brand identification characters;licensed characters;celebrities or popular personalities);

Nutrition/Health claims (e. g. nutrient claim, balanced, natural, diet or low-calorie);

Taste appeal (e. g. words to describe flavor or satisfied expression);

Emotional satisfaction (e. g. smiling or playing and use of the words ‘ fun’, ‘ happiness’ or ‘ pleasure’);

Culture (e. g. Chinese traditional festivals; patriotism);

Sports-related text (e. g. fuel, energy or strength).

### Data analysis

Data was categorized and encoded in Excel, then analyzed using SPSS 22·0. Categorical data was summarized as frequency (n) and percentage (%). The independent variables (IVs) of this study include setting type (with 3 levels: school surroundings, residential buildings, and public transit systems), urban–rural division (with 2 levels: central urban districts and peri-urban districts), and advertisement format (with 2 levels: video ads and print ads, applicable to residential buildings and public transit). The dependent variables (DVs) include the proportion of food ads (calculated as the number of food ads relative to the total number of ads in each setting), the proportion of unhealthy food ads (calculated as the number of unhealthy food ads relative to the total number of food ads in each setting), and the frequency of marketing themes (calculated as the occurrence count of specific marketing themes relative to the total number of unhealthy food ads in each setting or format). The comparisons were conducted using the χ^2^ test with a significance level of α = 0·05 (two-tailed) to examine group differences in DVs across different IV levels.

## Results

### Food advertisements around schools

#### Food advertisements by setting and display type and location

**Fig. 1:**
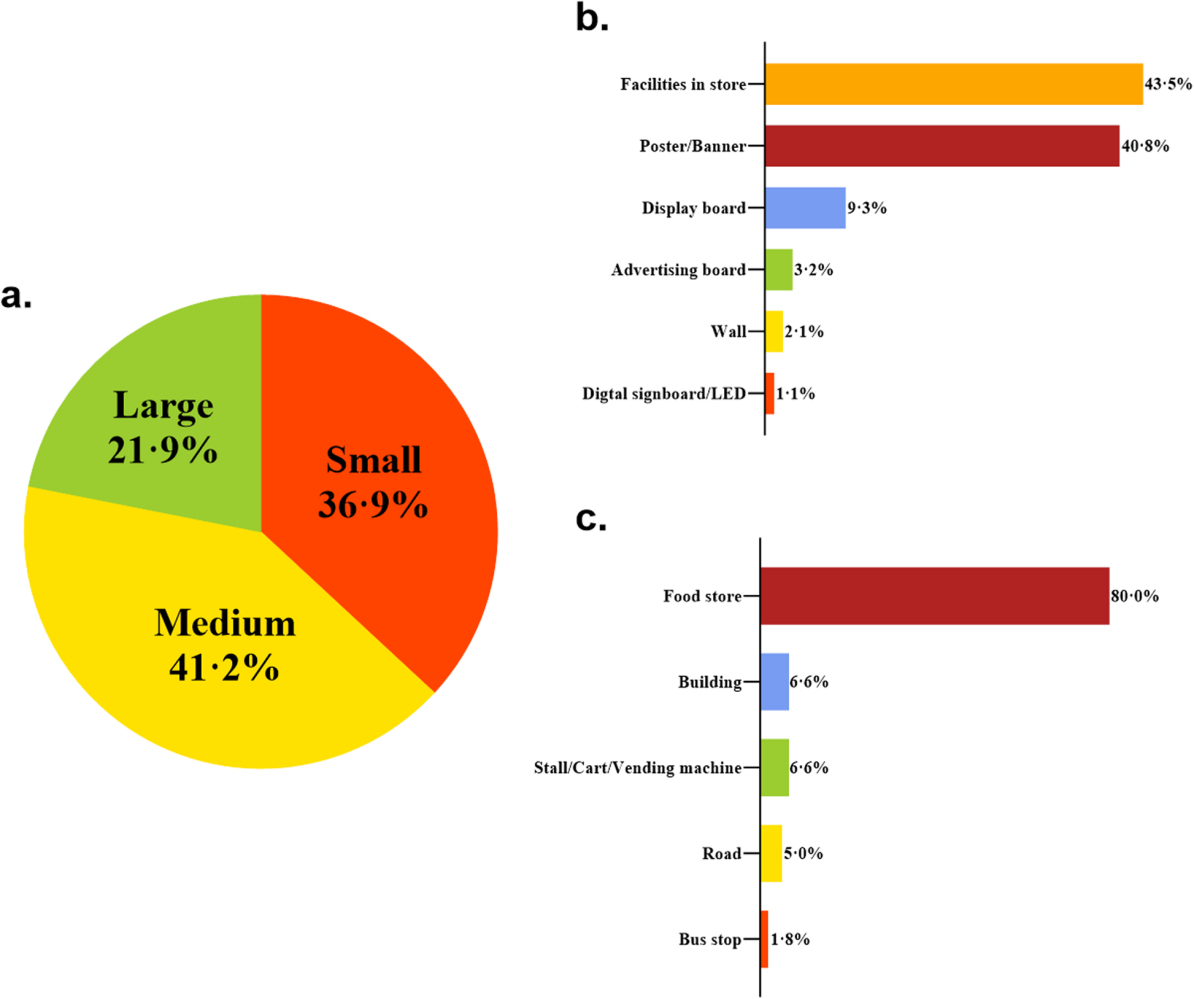
Distribution of outdoor advertisements around schools by setting, display type and location: size (**a**), display type (**b**), and location (**c**)

A total of 998 ads were identified around schools, with 439 (44·0%) of these being food-related ads. Medium and small-sized ads constituted the majority of food ads, representing 41·2% and 36·9%, respectively. The predominant formats of food ads included shop facilities (such as branded refrigerators, chairs, awnings, etc.) and posters/banners, which accounted for 43·5% and 40·8%, respectively. Furthermore, food ads were primarily situated near food shops, comprising 79·7% of the total. Refer to Fig. 1 for further details.

#### Types of advertised foods

**Fig. 2:**
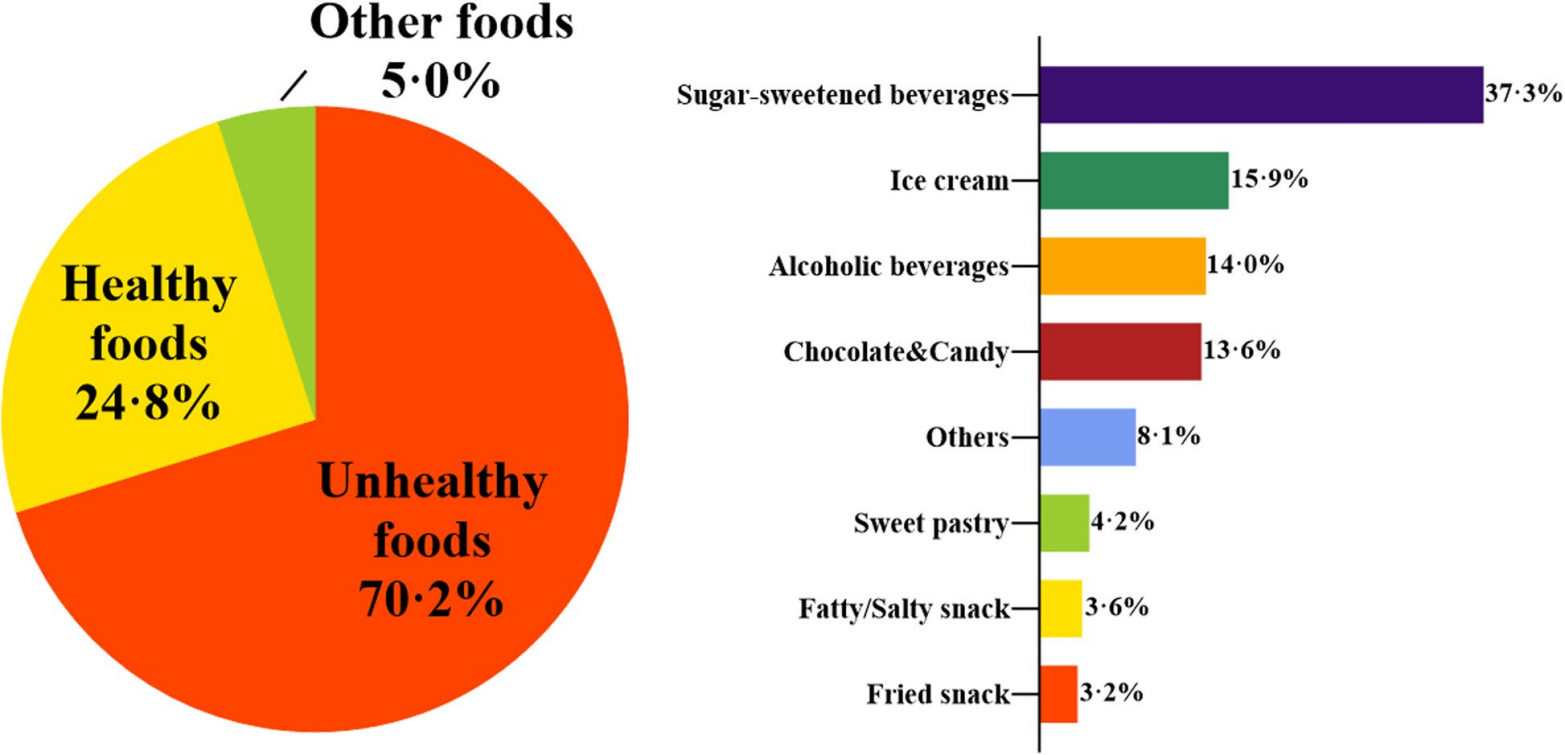
Distribution of food categories advertised around schools

Unhealthy food ads outnumbered those for healthy foods and other food categories, comprising 70·2%, 24·8%, and 5·0% of the total, respectively. The predominant types of food featured in unhealthy food ads included sugary beverages (37·3%), ice cream (15·9%), and alcoholic beverages (14·0%). For further details, please refer to Fig. 2.

### Food advertisements in residential buildings

#### Food advertisements by setting and display type

**Fig. 3:**
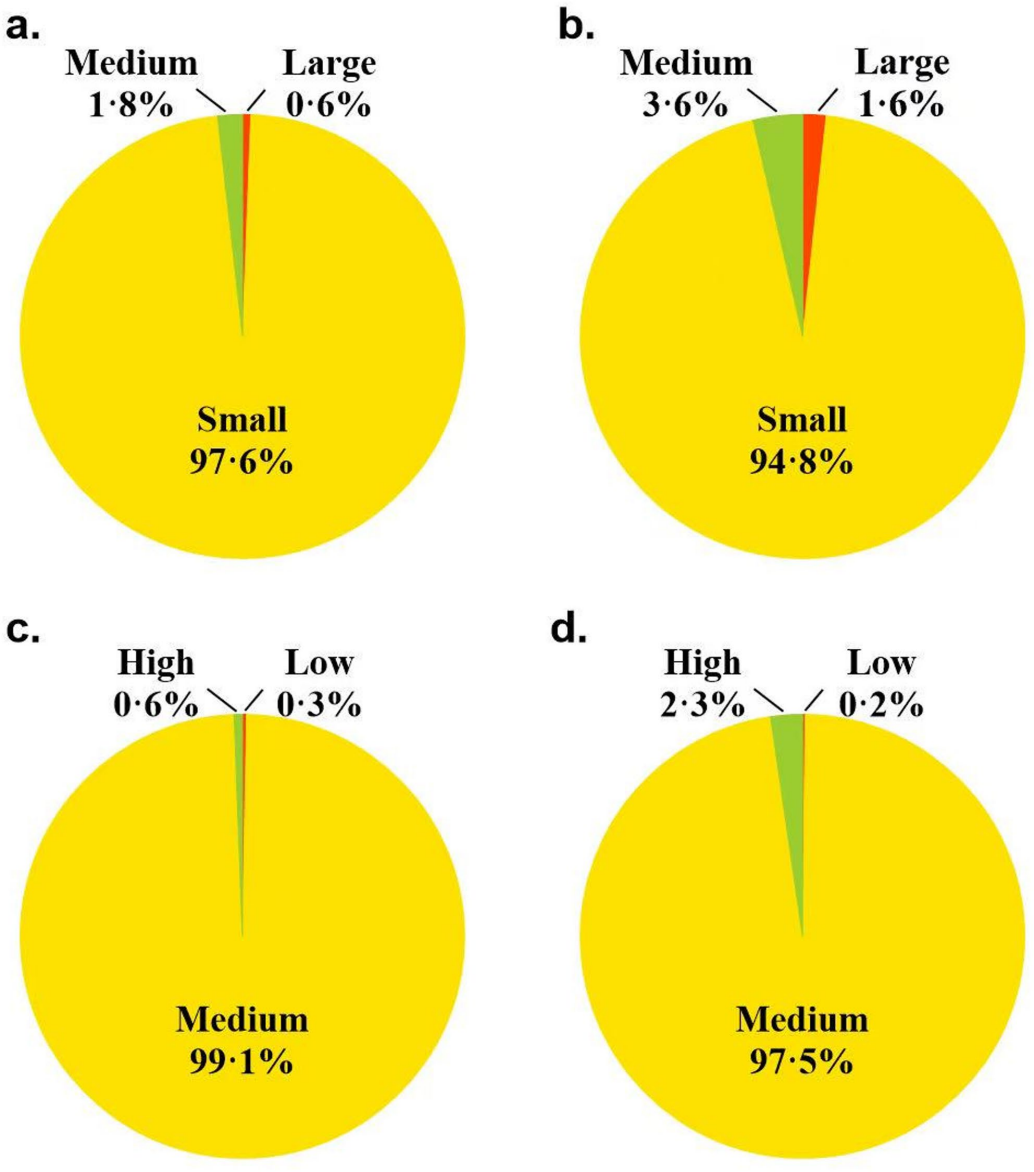
Size and height of outdoor advertisements in residential buildings: video ads (**a **& **c**) and print ads (**b **& **d**)

The survey revealed that elevator ads were present in 74% of all elevators. The research team collected a total of 4038 elevator ads in community buildings, including 1174 video ads (29·1%) and 2864 print ads (70·9%), excluding repetitive ads. Both types of ads were predominantly small-sized, accounting for 97·6% of video ads and 94·8% of print ads. Most of these ads were placed at a height of 0. 8 m to 1·8 m (99·1% and 97·5%, respectively), which was within the visible range of children. Refer to Fig. 3 for further details.

#### Types of advertised foods

**Fig. 4:**
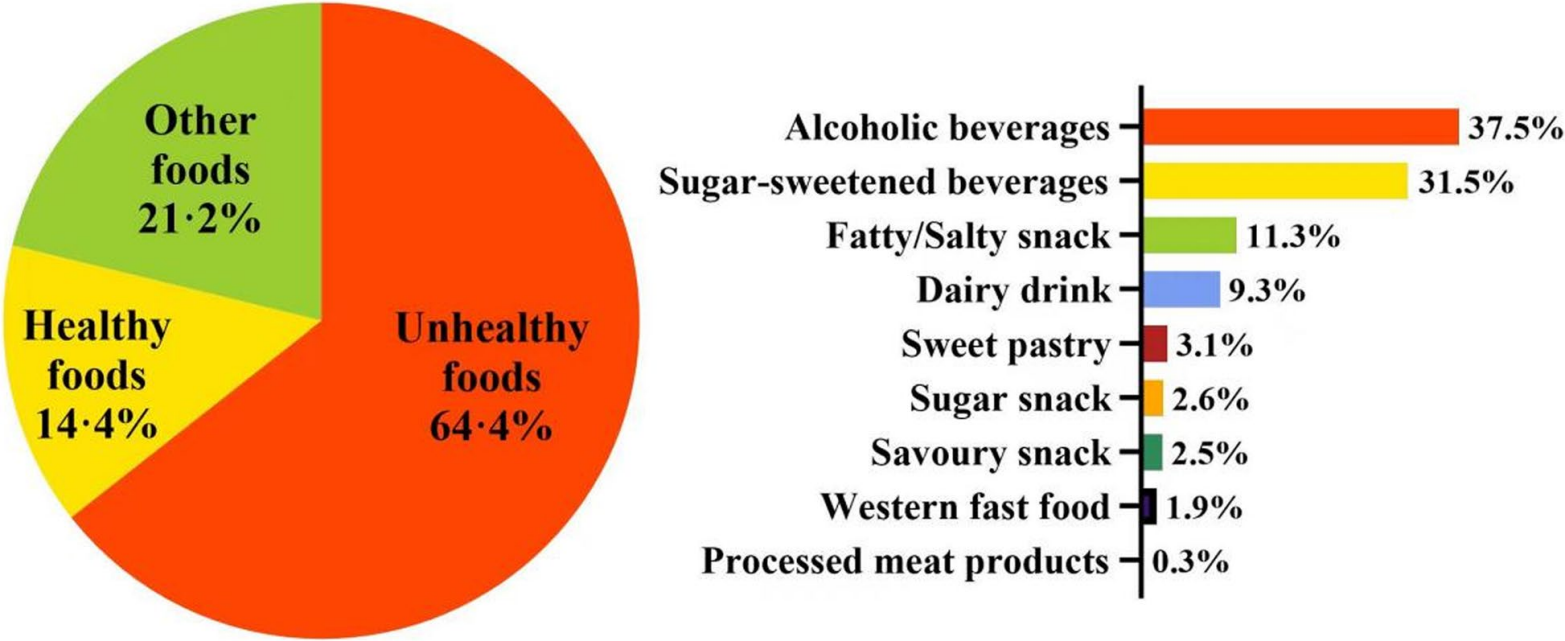
Distribution of types of food categories advertised (video) in residential buildings

**Fig. 5:**
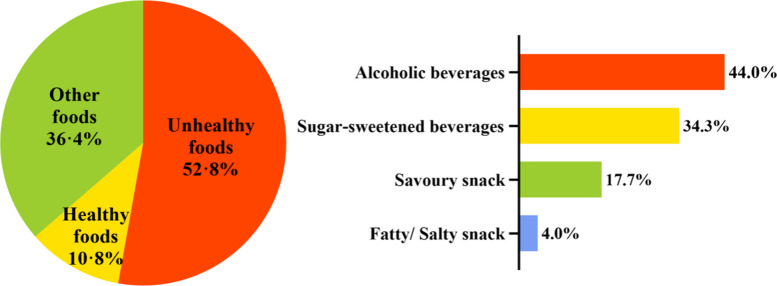
Distribution of types of food categories advertised (print) in residential buildings

Approximately one-third of both video and print ads were food-related (35·5% and 32·6%, respectively), with the majority promoting unhealthy foods (64·4% and 52·8%, respectively). The primary types of unhealthy foods in video ads were alcoholic beverages (37·5%) and sugary drinks (31·5%), while in print ads, there were also alcoholic beverages (44·0%) and sugary drinks (34·3%). For further details, please refer to Figs. 4 and 5.

### Food advertisements through public transit systems

#### Types of advertised foods

**Fig. 6:**
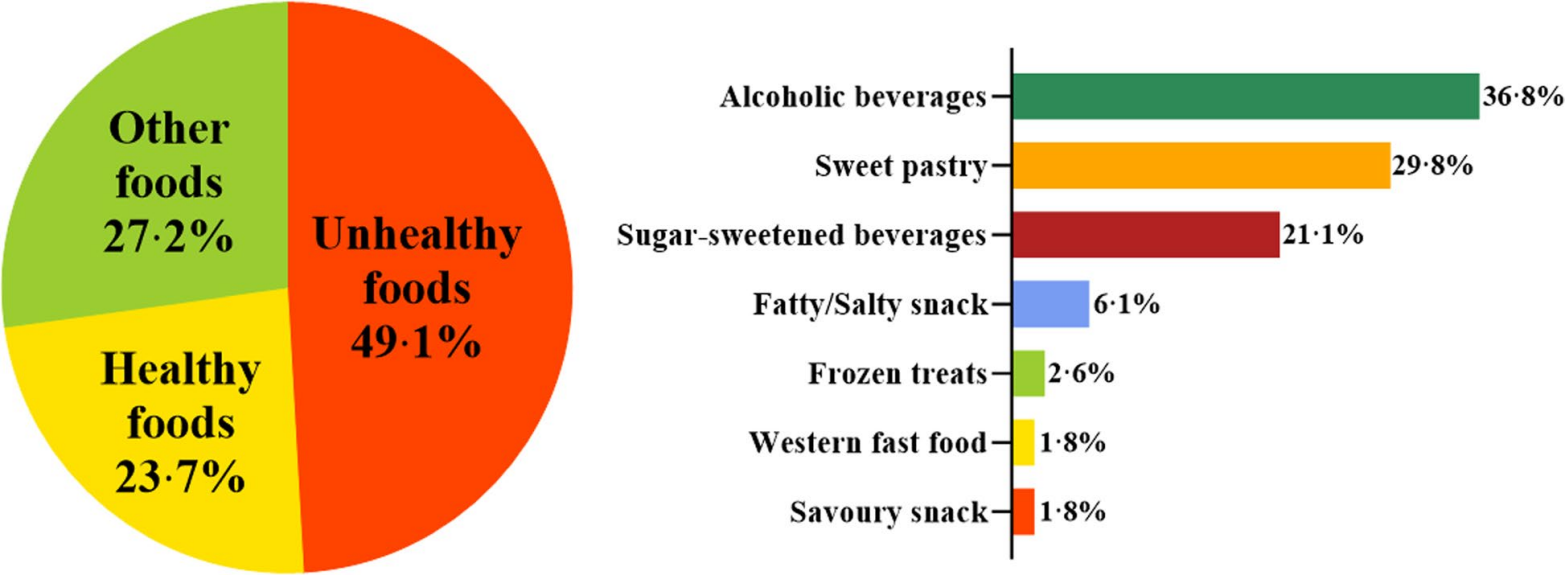
Distribution of food categories advertised through public transit systems

The survey identified a total of 3385 ads across transportation systems, of which 232 (6·9%) were food ads. Approximately half of these promoted unhealthy foods (49·1%). The primary types of unhealthy food ads included alcoholic beverages (36·8%), sweet pastries (29·8%), and sugary drinks (21·1%). See Fig. 6.

### Food advertisements by urban and peri-urban areas

The prevalence of print food ads in the elevators of residential buildings was markedly greater in peri-urban areas than in urban areas (54·9% vs. 24·7%, *P* < 0·05). Conversely, the proportion of food ads around schools, as well as video ads in the elevators of residential buildings, was greater in urban areas than in peri-urban areas (49·6% vs. 24·1% and 23·7% vs. 63·9%, *P* < 0·05). No significant differences were observed between the two regions regarding food ads in the public transit systems. For further details, please refer to Table 1.

**Table 1:** Distribution of food advertisements in urban and peri-urban areas [n (%)]

Area	Schools	Residential buildings (video) (s)	Residential buildings (print)	Public transit systems
Urban	386 (49·6%)	4859 (63·9%)	523 (24·7%)	157 (6·6%)
Peri-urban	53 (24·1%)	4371 (23·7%)	411 (54·9%)	75 (7·5%)
Total	439	9230	934	232
χ2	45·35	3793·56	229·81	0·85
P	< 0·05	< 0·05	< 0·05	0·36

A comparison of peri-urban and urban areas reveals that the proportion of unhealthy food ads surrounding schools was significantly higher in urban areas, with 72·8% of ads compared to 50·9% in peri-urban areas (*P* < 0·05). Similarly, video ads for unhealthy foods in the elevators of residential buildings were more prevalent in urban areas (73·8%) than in surrounding areas (53·9%, *P* < 0·05). However, no significant differences were observed between the two regions regarding print unhealthy food ads in the elevators of residential buildings and those in the public transit systems. For further details, please refer to Table 2.

**Table 2:** Distribution of unhealthy food advertisements in urban and peri-urban areas [n (%)]

Area	Schools	Residential buildings (video) (s)	Residential buildings (print)	Public transit systems
Urban	281 (72·8%)	3587 (73·8%)	285 (45·5%)	73 (46·5%)
Peri-urban	27 (50·9%)	2357 (53·9%)	202 (49·1%)	41 (54·7%)
Total	308	5944	487	114
^χ2^	10·63	397·38	2·64	1·36
*P*	< 0·05	< 0·05	0·11	0·24

### The marketing themes of unhealthy food advertising

Among the outdoor advertisements for unhealthy foods located near schools, the marketing themes most frequently employed included nutrition/health claims (7·1%), promotional characters (4·5%), and sports-related text (3·24%).

In print ads for unhealthy foods displayed in the elevators of residential buildings, premium offers was utilized at a rate of only 1·0%, while all other marketing themes were not utilized at all. Conversely, in the video ads for unhealthy foods within the same context, the most frequently used marketing themes were taste appeal (45·6%), nutrition/health claims (37·4%), and promotional characters (32·9%).

Among the ads for unhealthy foods through public transit systems, the most frequently used marketing themes were culture (28·1%), taste appeal (23·7%), and promotional characters (22·8%).

A comparative analysis revealed that video ads for unhealthy foods displayed in elevators of residential buildings used a greater variety of marketing themes. In contrast, the frequency of various marketing themes utilized in print ads for unhealthy foods in the elevators of residential buildings as well as around schools was significantly lower (*P* < 0·05). Furthermore, ads for unhealthy foods through public transit systems exhibited a notably high frequency of culture (*P* < 0·05), whereas the frequencies of other marketing themes were comparatively lower (*P* < 0·05). For further details, please refer to Table 3.

**Table 3:** Marketing themes of outdoor advertisements for unhealthy foods [n (%)]

Marketing Themes	Schools	Residential buildings (video) (s)	Residential buildings (print)	Public transit systems	^2^	*P*
Premium offers	6 (1·9)	525 (8·8)	5 (1·0)	1 (0·9)	61·87	< 0·05
Child-oriented features	5 (1·6)	850 (14·3)	0 (0·0)	0 (0·0)	137·00	< 0·05
Promotional characters	14 (4·5)	1955 (32·9)	0 (0·0)	26 (22·8)	333·39	< 0·05
Nutrition/health claims	22 (7·1)	2226 (37·4)	0 (0·0)	11 (9·6)	414·57	< 0·05
Taste appeal	0 (0·0)	2712 (45·6)	0 (0·0)	27 (23·7)	621·20	< 0·05
Emotional satisfaction	0 (0·0)	310 (5· 2)	0 (0·0)	2 (1·8)	45·98	< 0·05
Culture	0 (0·0)	1412 (23·8)	0 (0·0)	32 (28·1)	241·34	< 0·05
Sports-related text	10 (3·2)	904 (15·2)	0 (0·0)	2 (1·8)	133·07	< 0·05

According to the survey, the over all types of outdoor advertisements for unhealthy foods that require most attention are sugary beverages and alcoholic drinks. Among the outdoor advertisements for sugary drinks surrounding schools, the most commonly used marketing themes were nutrition/health claims (9·6%), sports-related text (7·0%), and promotional characters (4·3%). In the video sugary beverage ads in the elevators of residential buildings, the most frequently used marketing themes were nutrition/health claims (49·1%), sports-related text (45·8%), and promotional characters (43·6%). Regarding outdoor advertisements for alcoholic beverages near schools, the most commonly utilized marketing themes were nutrition/health claims (4·6%), premium offers (2·3%), and promotional characters (2·3%). In the video ads for alcoholic beverages found in the elevators of residential buildings, the marketing themes most frequently observed were taste appeal (77·3%) and cultural references (63·4%).

Compared to the areas surrounding schools, video ads for sugary beverages displayed in the elevators of residential buildings utilized marketing themes with greater frequency, except for emotional satisfaction and child-oriented features (*P* < 0·05). Additionally, the frequencies of marketing themes related to taste appeal and culture in video ads for alcoholic beverages in the elevators of residential buildings also exceeded those found in school-adjacent areas (*P* < 0·05). Overall, it can be observed that a greater variety of marketing themes were employed in video ads for both sugary drinks and alcoholic beverages within the elevators of residential buildings. For further details, please refer to Table 4.

**Table 4:** Marketing themes of the advertisements for sugar-sweetened beverages and alcoholic beverages around schools and in residential buildings [n (%)]

Marketing Themes	Sugar-sweetened beverages				Alcoholic beverages			
	Schools	Residential buildings (video) (s)	^χ2^	*P*	Schools	Residential buildings (video) (s)	^χ2^	*P*
Premium offers	1 (0·9)	260 (13·9)	16·05	< 0·05	1 (2·3)	0 (0·0)	··	0· 2
Child-oriented features	0 (0·0)	25 (1·3)	1·55	0·21	0 (0·0)	15 (0·7)	··	0·75
Promotional characters	5 (4·3)	818 (43·6)	68·86	< 0·05	1 (2·3)	432 (19·4)	0·45	0·5
Nutrition/health claims	11 (9·6)	922 (49·11)	68·18	< 0·05	2 (4·6)	15 (0·7)	··	0·04
Taste appeal	0 (0·0)	168 (9·0)	11·25	< 0·05	0 (0·0)	1722 (77·3)	137·73	< 0·05
Emotional satisfaction	0 (0·0)	10 (0·5)	0·62	0·43	0 (0·0)	285 (12·8)	6·30	0·01
Culture	0 (0·0)	0 (0·0)	··	··	0 (0·0)	1412 ((63·4)	72·13	0·05
Sports-related text	8 (7·0)	859 (45·8)	66·47	< 0·05	0 (0·0)	45 (2·0)	··	1·00

## Discussion

Food advertising regulation policies involve multiple stakeholders, including governments and businesses. Therefore, policy development requires a careful balance between effectiveness and feasibility. In its Framework for Implementing the Recommendations on the Marketing of Food and Non-Alcoholic Beverages to Children19, the WHO emphasizes that countries or regions that have not yet implemented relevant policies should take into full account the current advertising landscape, both domestically and internationally. Special consideration should be given to restricting the timing, placement, and types of foods featured in ads. Food companies often concentrate their advertising along children’s daily activity routes, especially around schools, leveraging “path dependency” to increase the likelihood of product purchases.This practice is common in countries with weak advertising regulations [23], where companies influence the child population through high-frequency, low-cost advertising strategies.

### Exposure of school-age children to unhealthy food advertisements in different settings in Chengdu

Our survey of the areas surrounding 25 schools revealed that unhealthy food ads are widespread in the vicinity of schools. Within a 250-m radius of each school, there were a total of 439 food ads. On average, there were 17 food ads around each school, the vast majority (70·16%) of which promoted unhealthy foods. Transforming the areas surrounding schools into “health-promoting zones” instead of “consumption-inducing zones” may contribute to curbing the global spread of childhood obesity to some extent. To address this issue, some countries have implemented measures such as establishing “ad-free buffer zones” around schools [24, 25]. In China, however, existing laws and guidelines are not sufficient to create “health promoting zones”. The *Advertising Law of the People’s Republic of China* (revised in 2021) does not explicitly prohibit the placement of unhealthy food ads in locations such as schools or public transportation areas. The *Law on the Protection of Minors* (revised in 2021) merely prohibits the broadcasting, posting, or distribution of commercial ads within schools [26]. In chengdu, The *Special Plan for Outdoor Advertising in the Central Area of Chengdu* designates areas such as administrative offices and educational and research institutions as zones where advertising is prohibited [27]. Additionally, the *Sichuan Province Food Safety Regulations*, which came into effect in 2023, prohibit food vendors from operating within 50 m of school entrances [28]. However, these regulations lack specific provisions regarding permanent food retail outlets near schools. Field observations around the surveyed schools revealed that the majority of food ads were located outside grocery stores (79·95%) and were frequently integrated into store infrastructure such as refrigerators, trash bins, chairs, umbrellas, and awnings (43·51%). Almost all the refrigerators and freezers used for food storage displayed outside the front door of the shop are covered with food ads. These ads were typically small to medium-sized, clearly visible, and prominently displayed. Despite their ubiquity and visibility, such forms of advertising are not currently regulated under existing laws and policies.

Elevator advertising, an efficient form of outdoor media, is experiencing continuous growth in market size worldwide. In China [29], elevator media has become a major channel for urban advertising and marketing. Similarly, in regions such as Europe, Latin America, and the Asia–Pacific, the elevator advertising market is also showing rapid growth [30], suggesting it should be considered a new focus for regulating the marketing of unhealthy foods. Elevator video ads are characterized with close-range exposure, repetitive playback, and compulsory viewing [31]. Children living in high-rise apartment buildings are inevitably exposed to elevator ads at a high frequency. Previous international research has rarely addressed residential elevator advertising, and this study is the first to focus on this everyday environment that is closely connected to children.

In residential buildings surveyed in Chengdu, over 30% of elevator ads fell into the category of food advertising. Among these, more than half were for unhealthy foods, and all elevator ads were placed within the visible range of children. A study on the exposure of children and adolescents in Beijing to pre-packaged food ads on television found that food ads accounted for 32·83% of all TV commercials, with unhealthy food ads making up 46·49% of that total [32]. In comparison, there is a higher proportion of unhealthy food ads in residential buildings. These findings suggest that children in urban China are significantly exposed to unhealthy food advertising while waiting for and riding residential elevators. In August 2022, Chengdu implemented China’s first group standard for elevator media advertising [33], which regulates the placement, quantity, size, and volume of elevator ads. However, there are still no specific restrictions regarding the content of these ads.

Public transit systems are the primary means of commuting for school-aged children in China, and public transit advertising constitutes a significant segment of outdoor advertising. In public transit systems of Chengdu, the majority of ads are public service announcements. Among the commercial ads, food ads account for only 6·9%, which is significantly lower than the proportions found around schools (43·99%) and in residential buildings (32·61%), as well as lower than the averages of many other countries, such as Northern England, [34] Sydney, [35] and so on. Consequently, school-aged children in Chengdu experience less exposure to unhealthy food advertising during their commutes, an outcome attributed to the city government’s active promotion of public service ads in transit spaces. The government uses the public transit network to promote the city’s image. In 2016, Chengdu removed all commercial vehicle body ads, retaining only 300 buses for public service advertising. In 2021, the city launched 15 metro trains themed around public welfare campaigns. However, consistent with other research, a significant portion—almost half—of the food ads that do appear on public transit of Chengdu still promote unhealthy foods.

### Sugar-sweetened beverages and alcoholic beverage advertisements are the most common types of unhealthy food advertising

School-aged children in Chengdu are most frequently exposed to unhealthy food ads of sugar-sweetened beverages and alcoholic beverages. This high density of SSB advertising aligns with global trends: a scoping review of 21 middle- and high-income countries found that sugary drinks are the most commonly promoted unhealthy food in outdoor advertising [14], reflecting the industry’s focus on capturing child and adolescent consumers.Alcoholic beverages typically yield high profit margins, and advertising is the industry’s primary method of market promotion. Although China has implemented measures to restrict alcohol advertising targeting children and adolescents—such as the *Regulations on the Administration of Alcohol Advertising* (in effect since 1996), which governs alcohol ads in mass media like television, radio, newspapers, and magazines—these regulations do not cover outdoor advertising. The *Law on the Protection of Minors* (revised in 2021) explicitly prohibits the sale of alcohol to minors. However, certain alcohol ads in subway system of Chengdu, such as a liquor slogan reading “Tribute to Every Moment of Hard Work”, circumvent regulation through ambiguous wording. Furthermore, Chengdu is located in Sichuan Province, a region with a deep-rooted baijiu (Chinese liquor) culture. Baijiu is a pillar industry in Sichuan’s economy, and the province ranks among the highest in alcohol consumption in China. Government regulation of alcohol advertising thus tends to be relatively lenient. In the three surveyed settings, baijiu ads often employ culturally themed marketing strategies, such as the slogan “Sichuan Baijiu Reigns Supreme” to downplay health risks and avoid direct warnings. In developing countries and regions where alcohol advertising is poorly regulated, a key challenge remains: how to curb the impact of alcohol ads on children’s health, especially when such advertising is driven by strong economic interests.

### The density and types of outdoor food advertising in Chengdu exhibit a combination of characteristics typical of both developing and developed countries

Numerous studies have shown that the density of outdoor food advertising is closely related to a region’s socioeconomic status and population density. The relationship between overweight/obesity and socioeconomic status follows a similar pattern. On one hand, high-income countries tend to enforce strict regulations on food advertising. Since 2007, the UK has banned the broadcasting of ads for HFSS during children’s program time slots and the scope of the ban is gradually expanding in recent years [36]. Particularly in affluent communities, the prevalence of unhealthy food advertising is lower [37], with a trend toward the “elitization” of healthy food marketing, such as the promotion of whole grain products in high-income neighborhoods [38]. In these countries, including the United States, France, and Italy, overweight and obesity rates are negatively correlated with socioeconomic status and related indicators [39]. On the other hand, in low- and middle-income countries or regions, weak government regulation and the proliferation of low-cost advertising have led to a different landscape. In densely populated cities such as Jakarta [40] and Nepal [41], street hand-painted ads and mobile food cart ads predominantly promote cheap snack foods. Even in low-income communities within developed countries, there is a high prevalence of ads for cheap, high-calorie foods. For example, in the suburbs of Melbourne, food ads at bus stops are more likely to promote fast food chains [42]. In developing countries such as Iran, overweight and obesity rates are positively correlated with socioeconomic status and related indicators [43]. Moreover, regardless of whether in developed [44] or developing [45] nations, there exists a phenomenon of “advertising bombardment” in densely populated areas.

Although China is classified as a developing country, its rapid economic growth has placed it in a unique transitional stage. In urban core and suburban areas of Chengdu, a dual pattern emerges in the density and types of outdoor food advertising, reflecting characteristics of both developing and developed countries. In Chengdu, food outdoor advertisements, particularly those promoting unhealthy foods are primarily concentrated in the central city. Around schools in these central districts, both food advertising and unhealthy food advertising are more prevalent than in suburban areas. Similarly, the proportion of video (digital/video) food ads and unhealthy food ads in residential buildings is also higher in the urban core compared to the outskirts. This indicates that in China, school-aged children living in areas with higher population density and relatively higher economic status are exposed to more outdoor advertising for unhealthy foods. At the same time, a higher proportion of print food ads is observed in suburban Chengdu compared to the city center. Advertisement cost is a key consideration for advertisers. As a traditional low-cost advertising format, print ads are more suitable for deployment in areas with lower population density and lower economic levels.Governments around the world should pay close attention to the issue of unhealthy food outdoor advertising in densely populated areas and implement appropriate regulatory measures.

### Nutrition/Health claims have increasingly become a primary marketing theme for unhealthy food advertising

WHO, in its *Set of Recommendations on the Marketing of Foods and Non-alcoholic Beverages to Children*, states: “The overall policy objective should be to reduce both the exposure of children to food marketing and the power of such marketing.” The WHO emphasizes that food marketing and communication activities involve two core elements: the choice of communication channels and the creation of marketing messages. Among these, the content of the marketing message, particularly the persuasive creative themes used affect the “power” of the marketing, or the extent to which the message achieves its intended impact [46]. When food companies, retailers, and other stakeholders use persuasive marketing themes to promote unhealthy foods and beverages to children, they increase the likelihood of children purchasing or consuming the advertised products and influences their dietary preferences [47]. This highlights the importance of restricting the use of persuasive marketing techniques in ads for unhealthy foods. However, due to the lack of a standardized definition and classification of marketing power, especially in terms of what makes marketing appealing to children. There is currently a gap in research on the power of outdoor food advertising. In the limited studies available, the most common persuasive advertising themes identified include premium offers, promotional characters, nutrition/health claims, taste appeal, and emotional satisfaction [48], similar to the strategies identified in television food marketing [49].

Within “residence–public transit system–school” environments of Chengdu, we observed two main media formats for outdoor food advertising: print ads (distributed across all settings) and video ads (primarily located in residential buildings and inside public transportation vehicles). Different media formats have distinct characteristics and target audiences, leading to variations in the use of advertising themes, with video ads tending to employ a wider range of marketing themes more frequently. Around schools, where print ads dominate, fewer marketing themes are used. This may be due to the strict monitoring of the food safety environment around schools by the Chengdu government. These ads mainly rely on their strategic geographic location to directly showcase the product, minimizing the need for elaborate marketing themes. Most unhealthy food ads in school surroundings use bold text or striking images to display the product name and visuals, emphasizing visual impact. The most common marketing theme observed was nutrition/health claims (7·14%), where ads use phrases like “low energy, no burden” to promote the supposed “healthiness” of unhealthy foods. Within public transit systems, unhealthy food ads employed six different marketing themes. Due to strict regulations on advertising in the transit systems, only culture appeared with slightly higher frequency. In contrast, video ads in residential buildings utilized all eight identified marketing themes, and their frequency was significantly higher than those observed around schools or in the public transit systems. Similar to television commercials, video ads can convey messages through both visual and auditory channels, allowing for the incorporation of a greater number of marketing themes to capture the audience’s attention. In elevator waiting areas or confined elevator spaces, where viewing time is brief, the ads instead feature catchy slogans and visually engaging content to quickly attract interest. The most commonly used marketing themes in these video ads are taste appeal (45·6%), nutrition/health claims (37·4%), and promotional characters (32·9%).

Then, we conducted a comparison of the marketing themes used in sugar-sweetened beverages ads and alcoholic beverages in the areas around schools and in residential buildings. Sugar-sweetened beverages ads across both settings and alcoholic beverages ads around schools commonly employed nutrition/health claims, sports-related text, and promotional characters as their primary marketing themes.

Nutrition/health claims are traditionally designed to persuade adults, but they are increasingly appearing in ads targeting children [50]. Food companies often portray sugar-sweetened beverages as “healthy choices” by emphasizing a single nutrient (e.g., “rich in vitamin C” fruit drinks), using vague functional claims (e.g., “aids digestion” in probiotic beverages), or making “natural” claims (e.g., sugar-free versions of sugar-sweetened beverages). School-aged children generally lack the ability to critically assess the truthfulness of such claims, which can lead to misconceptions about what constitutes a healthy food. This, in turn, can influence both their own and their parents’ purchasing decisions. Children may mistakenly equate consuming “advertised healthy foods” with healthy habits or associate the concept of “health” with specific brands. To some extent, this contradicts the original intent of health claims, which is to promote informed and healthy choices. Similarly, sports-themed outdoor food ads associate sugar-sweetened beverages with positive images such as health, energy, and vitality, which can significantly influence children’s food choices and long-term health outcomes. Children often overestimate the amount of energy burned through physical activity, leading to excess calorie intake when consuming advertised products. Conversely, when children engage in physical activity to earn or justify consuming such products, it can undermine their intrinsic motivation for healthy behaviors. Moreover, the portrayal of “ideal body types” in sports-related ads may trigger body image anxiety among older children, potentially resulting in picky eating, loss of appetite, or even eating disorders.

The use of these strategies [51] is becoming increasingly common in middle- and high-income countries, posing new challenges for advertising regulation. Chile’s Law of Food Labeling and Advertising, implemented in 2016, has adopted a strict, legislation-based regulatory approach. For example, it mandates warning labels on the front of packaging for unhealthy foods and beverages, and prohibits the use of “health” claims on the packaging and ads of high-sugar or high-fat products [52]. France requires that all food ads, whether targeting children or adults, across all forms of media provide relevant nutritional information if the product is processed or contains sugar, salt, or fat levels that exceed standard limits [53]. These regulatory measures are worth learning from for other countries. Regulating advertising content by requiring ads to simultaneously display the overall nutritional information of the product, setting up public service ads promoting “real sports nutrition” in sports venues (such as showing athletes drinking water instead of soda), and similar initiatives are all feasible management approaches. In addition, it is recommended to incorporate modules on proper nutrition and scientific exercise into school curricula. By promoting health literacy education, children’s awareness of health can be improved, helping to foster the value that “exercise is for health, not for snacks”.

Drawing on international experience in regulating unhealthy food advertising, and taking into account the specific context of Chengdu, the following measures can be considered: (1) Food Classification System: The Chinese Nutrient Profiling Model released in 2024 by the Chinese Nutrition Society [54] provides a health-based food classification standard tailored to China’s national conditions. It can be used to mandate clear labeling of unhealthy foods ads, similar to the UK’s [55] “traffic light” front-of-pack (FOP) labeling system or the pilot “Nutrition Choice” beverage classification system in Shanghai [56]. (2) Designating Ad-Free Zones for Unhealthy Foods: Establish areas where unhealthy food advertising is prohibited, such as within 100 or 200 m of schools. Ads for sugary drinks, alcoholic beverages, and high-fat foods should be banned in these zones. Grocery stores and similar establishments should also be restricted from displaying such ads again within these designated areas. (3) Regulating Residential Building Ads: Inspired by regulations on TV ads, manage unhealthy food advertising in residential buildings, for example, by restricting such ads during peak times when school-age children are commuting, limiting the total number of unhealthy food ads, and controlling the frequency of repeated broadcasts. (4) Health Advertisement Quota System: Introduce a quota system to increase the visibility of healthy food ads as a countermeasure to the appeal of unhealthy ones. For instance, require that 30% of advertising space near schools be allocated to nutrition education. Utilize elevator ads in residential buildings for health messaging, and develop “healthy buses” that incorporate health education into the daily commute of school-age children. These measures aim to integrate health awareness into every step of a child’s journey from home to school, ultimately creating a replicable “Chengdu Model” for childhood obesity prevention.

### Strengths and limitations

This study attempts to depict the exposure of school-aged children in Chengdu to outdoor advertisements for unhealthy foods, as well as the influence of these ads. In the site selection of this study, in addition to areas around schools and public transportation systems, Chinese school-aged children's actual daily environment was includedfor the first time, so outdoor advertisements in residential elevators were included in the survey scope. This approach fully covers the daily route of school-aged children, encompassing the “residence–public transportation system–school” chain. Nevertheless, this study only observes the distribution of outdoor advertisements for unhealthy foods in Chengdu through a cross-sectional approach. It does not investigate the relationship between the prevalence of overweight/obesity among school-aged children in Chengdu and their exposure to such ads, nor does it examine the impact of these ads on children’s dietary behaviors and preferences. Future research will aim to explore these connections further.

## Conclusion

Overall, the results suggest that the food advertising environment in Chengdu, especially in residential buildings and around schools, was not conducive to promoting healthy eating habits among school-age children. The high prevalence of ads for unhealthy foods, particularly sugar-sweetened beverages and alcoholic beverages, may increase children’s exposure to such products and influence their food preferences and consumption behaviors, recognized as a major contributing factor to childhood obesity and related noncommunicable diseases.

The findings have particular implications for policies that regulate outdoor advertising of unhealthy foods to improve childhood obesity. Although Chengdu has implemented some regulations regarding the placement of outdoor food ads near educational institutions, there is still a need for more comprehensive and effective policy to address this issue. Specifically, policies should focus on restricting the marketing of sugar-sweetened beverages and alcoholic beverages, given their high prevalence in outdoor advertisements and clear adverse effects on children’s health. In the meantime, it is crucial for relevant government departments, parents, and educational institutions to work together to enhance children’s nutrition and health education, creating a healthier food advertising environment for all children.

## Data Availability

Additional details in the form of a full study protocol are available by request to the corresponding author.

## Abbreviations

UNICEF: United Nations Children’s Fund
WHO: The World Health Organization
HFSS: Foods high in fat, sugar and salt
INFORMAS: The International Network for Food and Obesity/Non-communicable Diseases Research, Monitoring and Action Support

## Footnotes

1 a The Chinese Nutrition Society, in collaboration with UNICEF China, developed a Nutrient Profiling Model and launched it in July 2024 (after our study).
